# Supplementary material for: Quantifying contact status and the air-breakdown model of charge-excitation triboelectric nanogenerators to maximize charge density
Source: Nat Commun. 2020 Mar 27;11:1599. doi: 10.1038/s41467-020-15368-9 (PMC7101333; doi:10.1038/s41467-020-15368-9)
Supplement: Supplementary file 1 — Supplementary Information [file 41467_2020_15368_MOESM1_ESM.pdf]

Supplementary Information

Quantifying contact status and the air-breakdown model of charge-excitation triboelectric nanogenerators to maximize charge density

Liu et al.

Supplementary Figure 1. The physical model traditional contact separation TENG.
Supplementary Figure 2. The fabrication process and images of carbon gel electrode.
Supplementary Figure 3. The photograph of the deposited electrodes.
Supplementary Figure 4. The output performance of CE-TENG with different contact status formed by sandpaper-electrodes.
Supplementary Figure 5. The photographs of main TENG devices with six contact optimizations.
Supplementary Figure 6. The output charge density of CE-TENG with different external capacitor.
Supplementary Figure 7. SEM images of different dielectric film.
Supplementary Figure 8. The output voltage curve of the external capacitor.
Supplementary Figure 9. Effective charge output density accumulation curve of CE-TENG.
Supplementary Figure 10. The current density and voltage of the CE-TENG under different operation frequency.
Supplementary Figure 11. Possible output decreasing process of CE-TENG.
Supplementary Table 1. The systematical comparison of the contact status of different thickness dielectric films used in the experiment.
Supplementary Table 2. The components of main TENG devices with six contact optimizations.
Supplementary Table 3. The contact efficiency and estimated actual charge output density of six contact optimizations.
Supplementary Note 1. Maximum charge output density limited by air breakdown in excitation TENG.
Supplementary Note 2. The effect of contact status on output with reducing the dielectric thickness.
Supplementary Note 3. Estimating the actual charge density output of CE-TENG.
Supplementary Note 4. Analysis of the decrease in charge density in the stability test.

Supplementary Figures

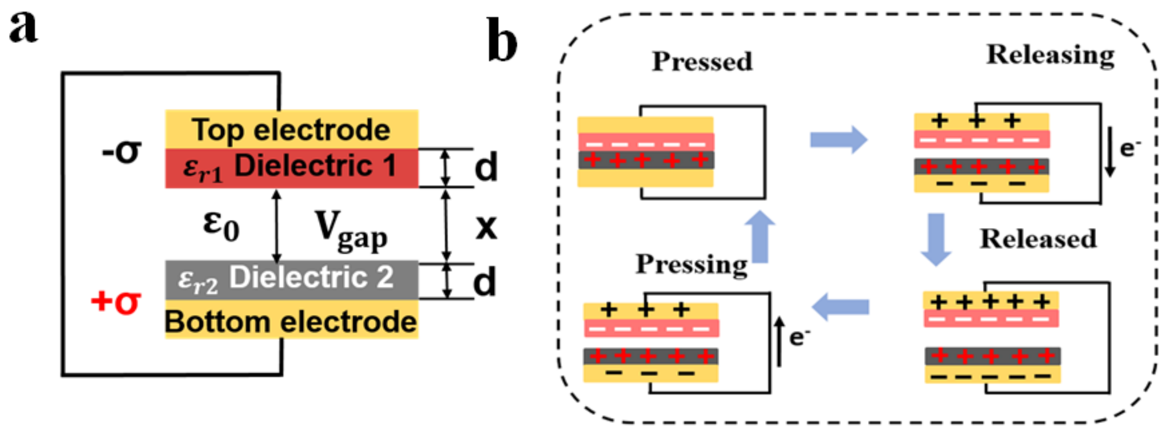

Supplementary Figure 1. The physical model traditional contact separation TENG. a, The physical and electric model of the traditional contact separation TENG and some critical parameters are also listed in the schematic. **b**, The working mechanism of the traditional contact separation TENG.

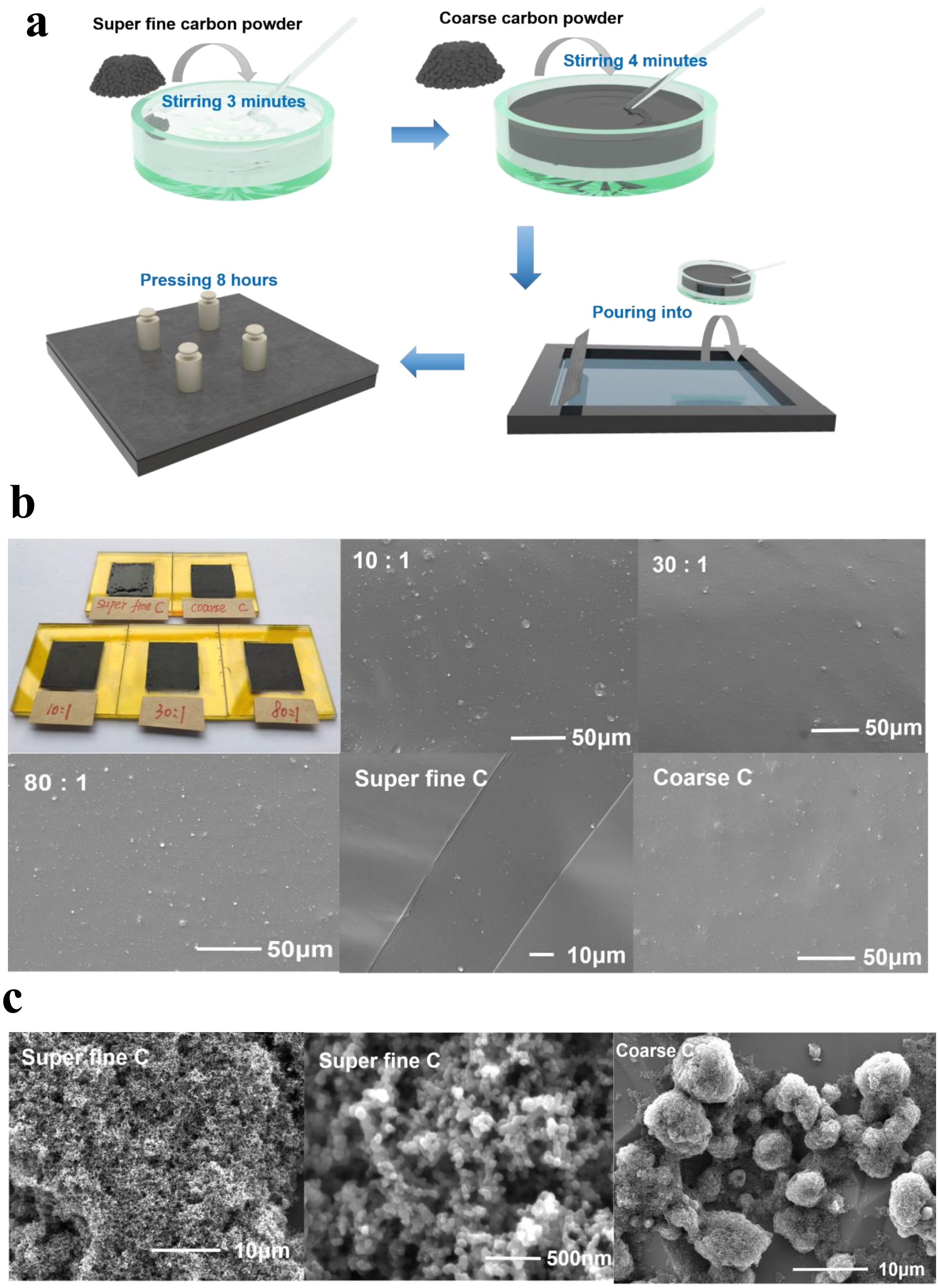

Supplementary Figure 2. The fabrication process and images of carbon gel electrode. a, The fabrication process of carbon gel electrode. **b,** The photo and SEM images of carbon gel electrodes with different proportions. **c,** The SEM images of coarse carbon powder and super fine carbon powder.

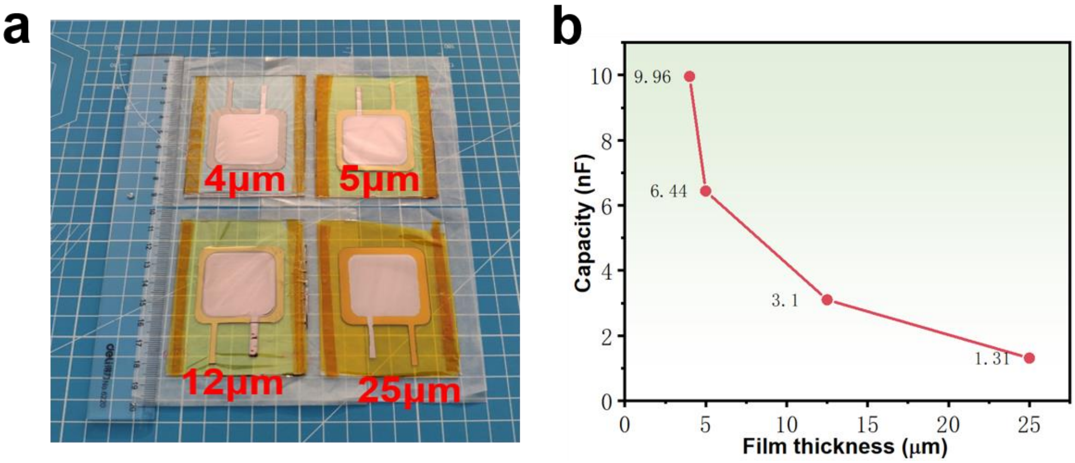

Supplementary Figure 3. The photograph of the deposited electrodes. a, The photograph of the deposited electrodes based on dielectric film with different thickness. **b**, The capacitance of the deposited electrodes based on dielectric film with different thickness(dielectric thickness: 4μm, 5μm,12μm,25μm).

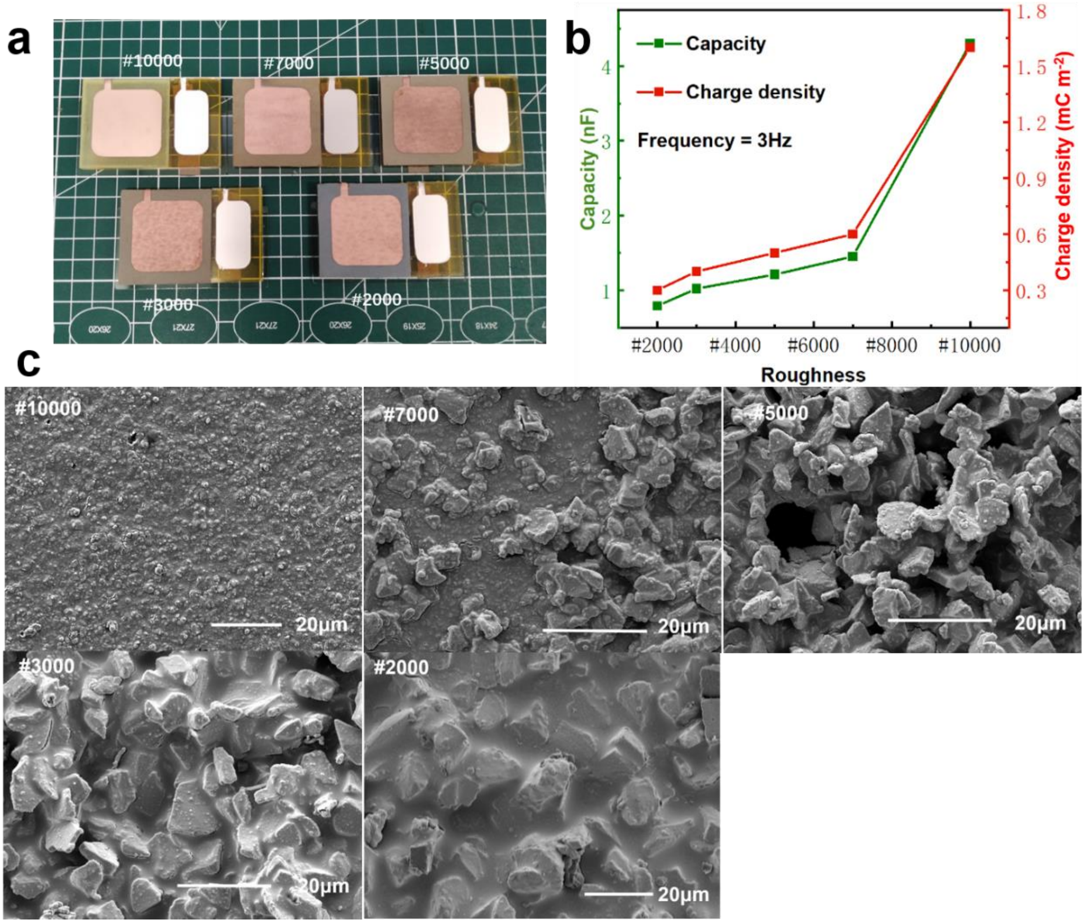

Supplementary Figure 4. The output performance of CE-TENG with different contact status formed by sandpaper-electrodes. a, The photograph of the deposited electrode based on sandpaper (electrode area: 3.2 × 3.2 cm^2). **b,** The output performance of the deposited electrodes based on sandpaper with different roughness. **(c)** SEM images of surface of deposited electrodes based on sandpaper with different roughness (#10000, #7000, #5000, #3000, #2000).

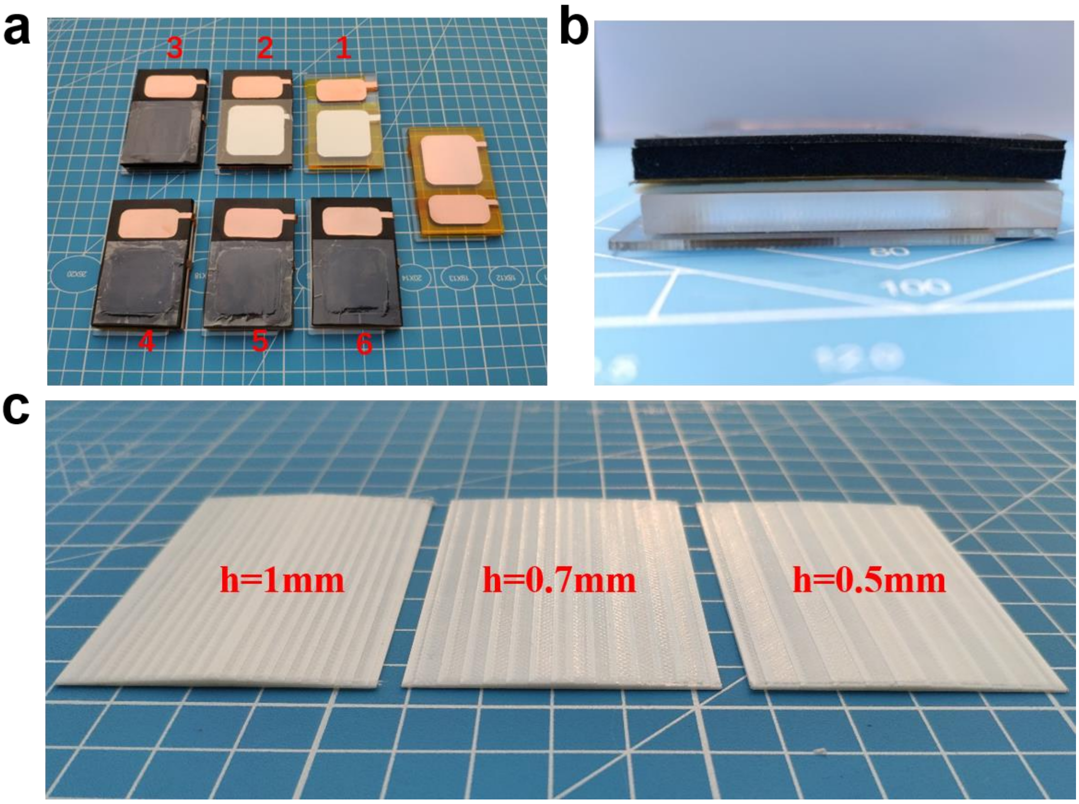

Supplementary Figure 5. The photographs of main TENG devices with six contact optimization. a, The photograph of the six kinds of devices which representative six different contact optimization (The structure composition see **Supplementary Table 2**). **b,** The side views of the device #6. **c,** The photographs of different thickness arch structure which is fabricated by 3D printer. (The “h” means the thickness of the raised part.)

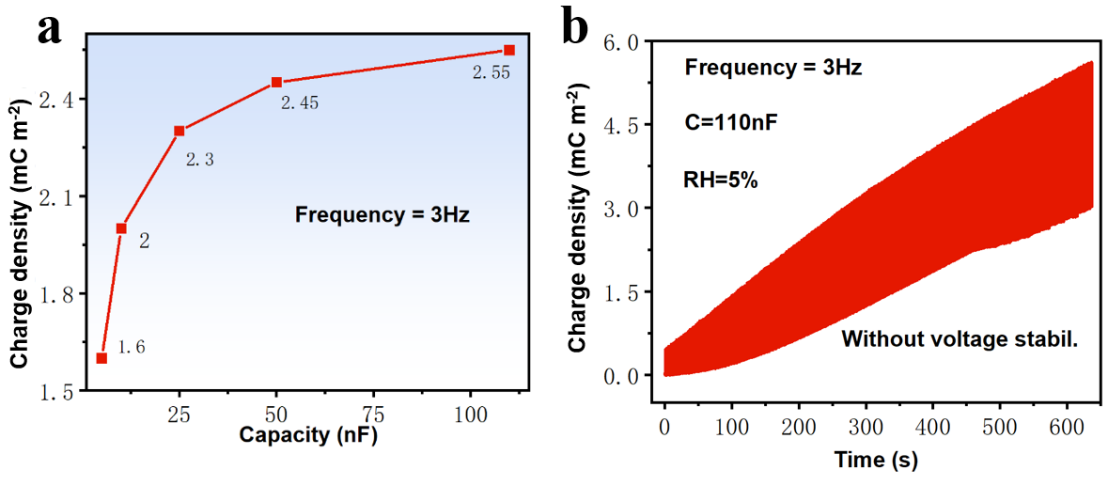

Supplementary Figure 6. The output charge density with different external capacity. a, The output charge density under different external capacitors without voltage stability, the working frequency of the CE-TENG is 3Hz. **b,** Dynamic charge density output of CE-TENG without using the zener diode (the maximum charge density can achieve 2.55 mC m^{-2} when the external capacitor is 110nF, the working frequency and the relative humidity are respectively 3Hz and 5%).

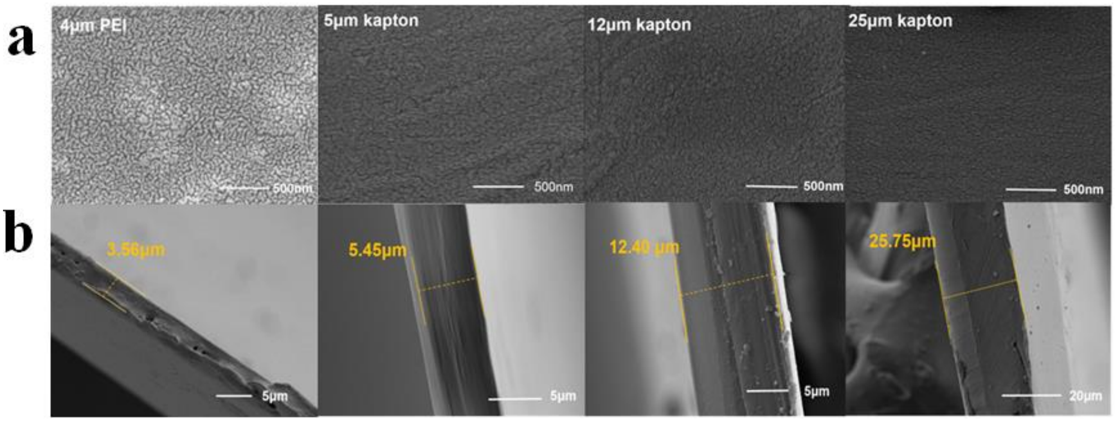

Supplementary Figure 7. SEM images of different dielectric film. a, SEM images of the surface of PEI film and kapton film with different measured thicknesses. **b,** SEM images of the thickness of PEI film and kapton film with different measured thicknesses.

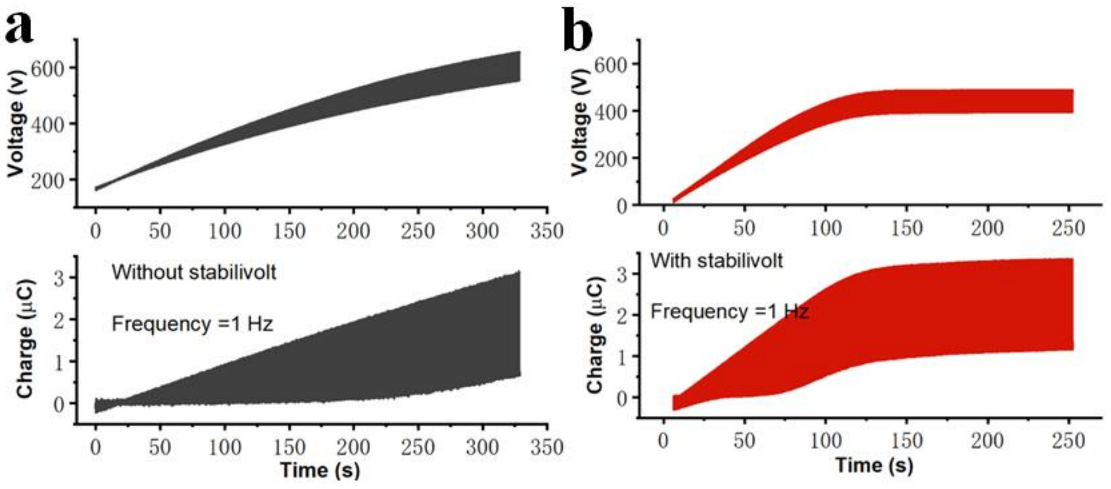

Supplementary Figure 8. The output voltage curve of the external capacitor. a, Voltage curve on external capacitor when working CE-TENG by external excitation TENG device without using Zener diode. **b,** Voltage curve on external capacitor when working CE-TENG by external excitation TENG device with using Zener diode (operation frequency: 1 Hz).

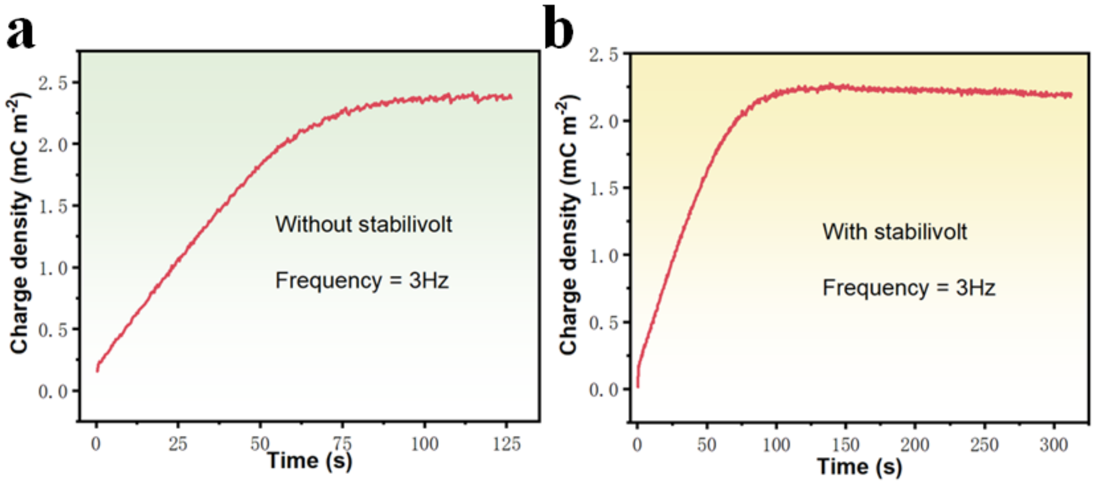

Supplementary Figure 9. Effective charge output density accumulation curve of CE-TENG. a, The output charge density accumulation curve of CE-TENG without zener diode. The maximum charge density can achieve 2.38 mC m^{-2}. **b,** The output charge density accumulation curve of CE-TENG with zener diode. The maximum charge density can achieve 2.25 mC m^{-2} (operation frequency: 3 Hz).

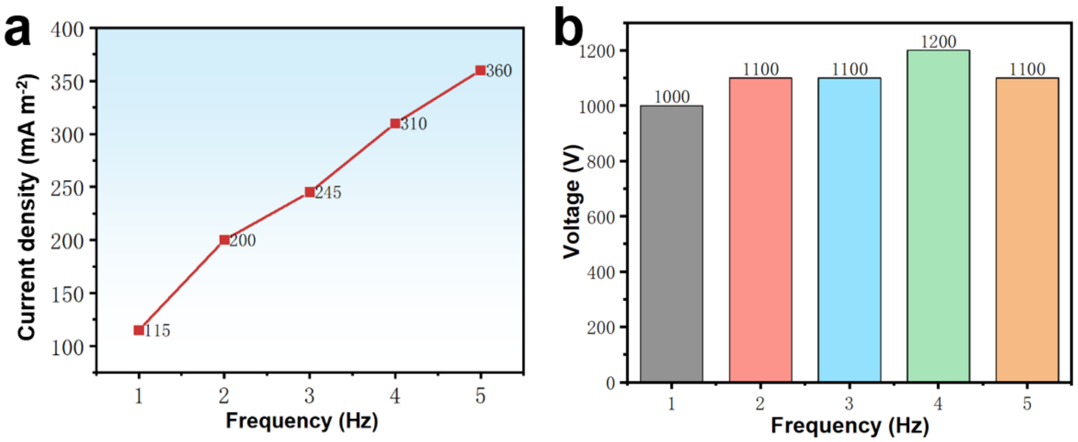

Supplementary Figure 10. The output current density and voltage of the CE-TENG under different operation frequency. a, The current density output of the CE-TENG with the device #5 under different operation frequency (1Hz, 2Hz, 3Hz, 4Hz, 5Hz). **b,** The load voltage of the CE-TENG with the device #5 under different operation frequency (1Hz, 2Hz, 3Hz, 4Hz, 5Hz).

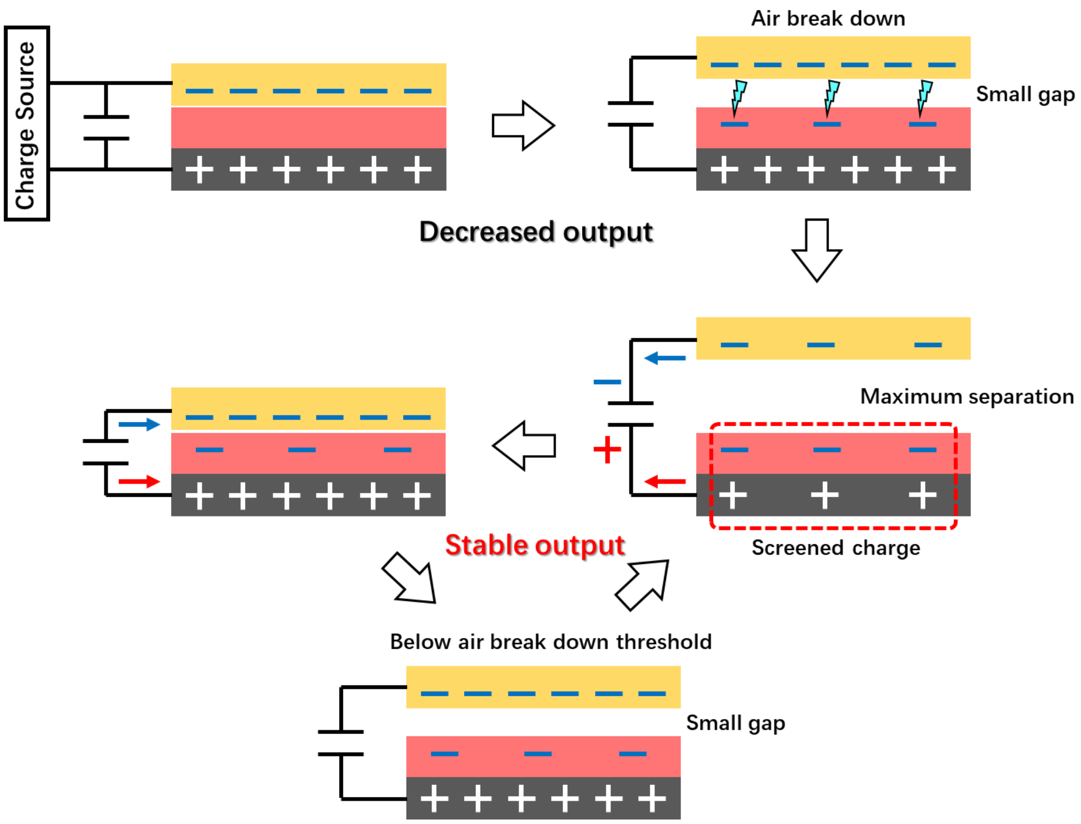

Supplementary Figure 11. Possible output decreasing process of CE-TENG. The air breakdown may bring about the decreasing of the output performance.

Supplementary Tables

Supplementary Table 1. The systematical comparison of the contact status of different thickness dielectric films used in the experiment

Film thickness	Deposited capacitance (C_0)	Contact capacitance (C_1)	Contact efficiency ($\eta = C_1/C_0$)	Traditional charge density	Actual charge density
4μm	9.96nF	5.48nF	54.98%	2.20 mC m^{-2}	4.001 mC m^{-2}
5μm	6.44nF	3.21nF	49.84%	1.55 mC m^{-2}	3.112 mC m^{-2}
12μm	3.10nF	2.01nF	64.84%	0.95 mC m^{-2}	1.466 mC m^{-2}
25μm	1.31nF	1.21nF	92.37%	0.60mC m^{-2}	0.649 mC m^{-2}

Supplementary Table 2. The components of main TENG devices with six contact optimizations

Device number	Acrylic base	Arch structure	Foam	Al electrode	Carbon gel electrode	Dielectric film
#1	√			√		√
#2	√		√	√		√
#3	√		√		√	√
#4	√	√	√		√	√
#5	√	√	√		√	√
#6	√	√	√		√	√

Supplementary Table 3. The contact efficiency and estimated actual charge output density of six contact optimizations (the area of electrode S_0 :10 cm^2)

Device number	Contact capacitance (C_1)	Contact efficiency (ŋ)	Actual contact area ($S=S_0$* ŋ)	Actual charge density (σ=Q/S)
#1	0.614nF	6.16%	0.616 cm^2	4.357 mC m^{-2}
#2	2.682nF	26.93%	2.693 cm2	3.085 mC m^{-2}
#3	4.579nF	45.97%	4.594 cm2	4.309 mC m^{-2}
#4	4.978nF	49.98%	4.998 cm2	4.322mC m^{-2}
#5	5.476nF	54.98%	5.498 cm2	4.001 mC m^{-2}
#6	5.036nF	50.56%	5.056 cm2	4.166 mC m^{-2}

Supplementary Notes

Supplementary Note 1. Maximum charge output density limited by air breakdown in excitation TENG

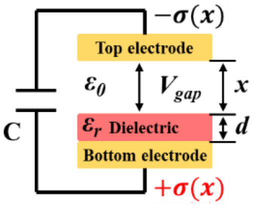

With charge exciting to TENG electrodes and according to Paschen's Law, air breakdown effect would occur between top electrode (TE) and the surface of dielectric primarily when the voltage (V_{gap}) in between exceeds a critical value during separation process. Therefore, deriving from V_{gap} and Paschen's Law in atmosphere condition, the maximum charge output density (δ_{max}) on parameters of dielectric layer can be obtained, which would have great significance in enhancing the output of charge excitation TENG (CE-TENG) from the intrinsic material aspect.

During the contact-separation process:

$$V_{TENG} = V_C \tag{1}$$

Where V_{TENG} is the voltage of TENG, V_C is the voltage of external capacitor.

When TENG gets fully contacted, the capacitance is:

$$C_{TENG}(0) = \frac{\varepsilon_0 \varepsilon_r S}{d} \tag{2}$$

Where S is the effective area of TENG and d is the thickness of dielectric. ε_0 and ε_r represent the vacuum permittivity and relative permittivity for dielectric material respectively.

In the contact state, we assume that the charge in TENG and external capacitor is Q and Q_C respectively. Thus, the following equation should be satisfied:

$$\frac{Q}{C_{TENG}(0)} = \frac{Q_C}{C} \tag{3}$$

$$Q = \sigma S \tag{4}$$

When the gap distance x>0, the capacitance of TENG under a random state can be expressed by:

$$C_{TENG}(x) = \frac{\varepsilon_0 S}{\frac{d}{\varepsilon_r} + x} \tag{5}$$

Meanwhile, at a random state, we assume the charge in TENG to be Q(x) and the charge in external capacitor would be $Q_c + (Q - Q(x))$. Therefore, the following equation should be satisfied as well:

$$\frac{Q(x)}{C_{TENG}(x)} = \frac{Q_C + (Q - Q(x))}{C} \tag{6}$$

According to Supplementary Equation 2-6, the charge and charge density in CE-TENG can be expressed by:

$$Q(x) = Q(1 - \frac{x}{\frac{d}{\varepsilon_r}+x+\frac{\varepsilon_0 S}{C}}) \quad (7)$$

$$\sigma(x) = \sigma\left(1 - \frac{x}{\frac{d}{\varepsilon_r}+x+\frac{\varepsilon_0 S}{C}}\right) \quad (8)$$

Therefore, the voltage of air gap can be expressed by:

$$V_{gap} = \frac{x\cdot\sigma(x)}{\varepsilon_0} = \frac{x\sigma}{\varepsilon_0}\left(1 - \frac{x}{\frac{d}{\varepsilon_r}+x+\frac{\varepsilon_0 S}{C}}\right) \quad (9)$$

According to Paschen's Law, the relationship of the breakdown voltage and the gap distance is:

$$V_{a-b} = \frac{A(Px)}{\ln(Px)+B} \quad (10)$$

Where P is the pressure of the gas, A and B are the constants determined by the composition and the pressure of the gas. For air at normal atmospheric pressure of 101 kPa, A is $2.87 \times 10^5\ V/(atm \cdot m)$, and B is 12.6.

In order to avoid air breakdown effect, the V_{gap} needs to remain smaller than $V_{a\text{-}b}$ at any $x > 0$ states. So the following relationship is needed:

$$V_{gap} \leq V_{a-b} \quad (11)$$

According to Supplementary Equation 8-11, the maximum charge density of CE-TENG can be expressed by:

$$\sigma_{max} = \left(\frac{AP\varepsilon_0}{(\ln(px)+B)\left(1-\frac{x}{\frac{d}{\varepsilon_r}+x+\frac{\varepsilon_0 S}{C}}\right)}\right)_{min} \quad (12)$$

Maximum charge density σ_{max} is affected by external capacitor and dielectric thickness, Which is different from that in common TENG.

When C approaches infinity: the voltage of air gap can be expressed by:

$$V_{gap,C\to\infty} = \frac{\delta dx}{\varepsilon_0(d+x\varepsilon_r)} \quad (13)$$

When C approaches infinity, the maximum charge density in TENG can be expressed by:

$$\sigma_{max,C\to\infty} = \left(\frac{AP\varepsilon_0(d+x\varepsilon_r)}{d(\ln(Px+B))}\right)_{min} \quad (14)$$

Obviously, when C approaches infinity, the maximum charge density of CE-TENG is equal to common TENG.

Supplementary Note 2. The effect of contact status on output with reducing the dielectric thickness

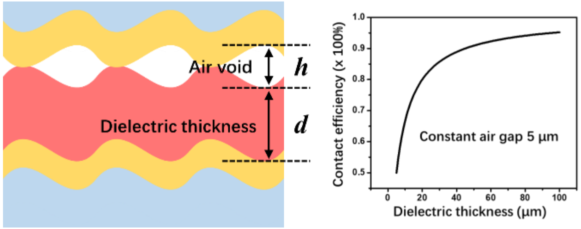

Here, we define the efficiency of contact status as the equation presented:

$$\eta = \frac{C_{contact}}{C_{film}} \tag{15}$$

Where $C_{contact}$ is the capacitance when device getting compressed, and C_{film} stands for the capacitance of dielectric film with deposited electrodes (Supplementary Figure 7). Considering the existence of air void under compressed state. The following equations can be obtained:

$$C_{contact} = \frac{\varepsilon_r \varepsilon_0 S}{\varepsilon_r h + d} \tag{16}$$

$$C_{film} = \frac{\varepsilon_r \varepsilon_0 S}{d} \tag{17}$$

Where S is the capacitor electrode area. ε_r and ε_0 is relative permittivity of dielectric and vacuum respectively. h represents the equivalent air gap created by air voids. d is the dielectric thickness.

Therefore, the efficiency of contact status can be expressed by:

$$\eta = \frac{d}{d + \varepsilon_r h} \tag{18}$$

According to Supplementary Equation 4, with a constant contact status (constant h), while reducing the dielectric thickness, the efficiency of contact would become worse. Supplementary Table 1 shows the experimental tested results.

Supplementary Note 3. Estimating the actual charge density of CE-TENG

In the experiment, although the contact status become better when using soft gel electrode, the capacitance under compressed state still has big loss compared with deposited electrode. In this case, herein, we would like to expect or estimate the charge density that can be achieved while realizing the 100% contact efficiency on TENG devices.

From the experimental test, we can obtain the capacitance of dielectric with deposited electrode (C_{film}) and each optimized devices ($C_{contact}$). Thus, the contact efficiency of each devices can be derived:

$$\eta = \frac{C_{contact}}{C_{film}} \quad (19)$$

Here, we define the actual contact area for TENG devices as S'.

$$S' = S_0 \times \eta \quad (20)$$

Where S_0 is the area of the electrode.

Therefore, the actual charge density can be estimated:

$$\sigma' = \frac{Q}{S'} = \frac{\sigma}{\eta} \quad (21)$$

Where, Q and σ is the tested output charge and output charge density.

In Supplementary Table 3 and Figure 7, we list the estimated actual contact area and actual charge density of 6 devices with contact optimizations.

Supplementary Note 4. **Analysis of the decrease in charge density in the stability test**

When the voltage provided by charge source become high enough, air break down would happen between the surface of top electrode and dielectric layer during separation process. In this process, the broken down charges would localized on the dielectric thus form a dielectric surface charge density. This kind of surface charge would form an electrostatic electric field between dielectric and bottom electrode so that to lock a partial energy which cannot be released while TENG device getting fully separated. Above process cause the output decrease effect. Afterwards, TENG got contacted again, and during the following separation process, although the voltage from charge source is high enough, air break down would not happen due to the existence of surface charge on dielectric surface that decrease the gap voltage. Therefore, during the following contact separation process, the output would become stable. All the processes are shown in Supplementary Figure 11.
