# Supplementary material for: Quantifying contact status and the air-breakdown model of charge-excitation triboelectric nanogenerators to maximize charge density
Source: Nat Commun. 2020 Mar 27;11:1599. doi: 10.1038/s41467-020-15368-9 (PMC7101333; doi:10.1038/s41467-020-15368-9)
Supplement: Supplementary file 3 — Description of Additional Supplementary Files [file 41467_2020_15368_MOESM3_ESM.pdf]

Description of Additional Supplementary Files

File Name: Supplementary Movie 1
Description: Mechanical property of carbon/silicone gel electrode.

File Name: Supplementary Movie 2
Description: Demonstration of charge curve without zener diode for CE-TENG.

File Name: Supplementary Movie 3
Description: Demonstration of charge curve with zener diode for CE-TENG.

File Name: Supplementary Movie 4
Description: Demonstration of lighting green LEDS for CE-TENG.

File Name: Supplementary Movie 5
Description: Demonstration of charging 2.2μF capacitor for CE-TENG.

File Name: Supplementary Movie 6
Description: Demonstration of powering thermos-hygrometer for CE-TENG.
